# Incidence and patient-related risk factors for external ventricular drain-related cerebrospinal fluid infections

**DOI:** 10.1016/j.bas.2026.105998

**Published:** 2026-03-02

**Authors:** Aron Alakmeh, Mergime Maralushaj, Vittorio Stumpo, Daniel de Wilde, Erik Edström, Adrian Elmi Terander, Stefanos Voglis, Flavio Vasella, Luca Regli, Carlo Serra, Giovanna Brandi, Victor E. Staartjes

**Affiliations:** a Machine Intelligence in Clinical Neuroscience & Microsurgical Neuroanatomy (MICN) Laboratory, Department of Neurosurgery, Clinical Neuroscience Center, University Hospital Zurich, University of Zurich, Zurich, Switzerland; b Neurosurgical Intensive Care Unit, Institute of Intensive Care Medicine, University Hospital Zurich, University of Zurich, Zurich, Switzerland; c Department of Clinical Neuroscience, Karolinska Institutet, Stockholm, Sweden; d Capio Spine Center Stockholm, Löwenströmska Hospital, Upplands Väsby, Sweden; e Department of Medical Sciences, Örebro University, Örebro, Sweden

**Keywords:** External ventricular drain, Cerebrospinal fluid, Infection, Risk factors, Ventriculostomy, Incidence

## Abstract

**Introduction:**

Despite procedural standardization, external ventricular drain-related infections (EVD-RI) remain frequent, with reported incidences of 5–30%. The underlying risk factors, however, remain insufficiently understood and have been debated for decades.

**Research question:**

To address this, patient-related and periprocedural risk factors for EVD-RI in adults undergoing EVD placement were analyzed.

**Materials and methods:**

This single-center cohort included adults receiving EVD implantation between 2014 and 2022. Suspected EVD-RI was defined according to Infectious Diseases Society of America (IDSA). Confirmed EVD-RI required a positive CSF culture and/or broad-range eubacterial PCR. The primary outcome was suspected EVD-RI. Patient-related factors were assessed for their role as independent predictors or confounders using a multivariable Cox proportional hazards model with purposeful variable selection.

**Results:**

A total of 387 patients with 421 EVDs were included. Suspected EVD-RI occurred in 104 (26.9%) patients and confirmed EVD-RI in 63 (16.3%) patients. EVD exchange (HR: 3.13, 95% CI: 2.02-4.85, *p* < 0.001), aneurysmal subarachnoid hemorrhage (aSAH) (HR: 2.56, 95% CI: 1.40-4.68, *p* = 0.002), and non-aneurysmal SAH (HR: 2.11, 95% CI: 0.90-4.96, *p* = 0.09) were associated with a significantly higher risk, whereas postoperative systemic antibiotics given for other suspected infections (HR: 0.33, 95% CI: 0.21-0.51, *p* < 0.001) and additional implantation of neuromonitoring (HR: 0.54, 95% CI: 0.30-0.97, *p* = 0.04) were associated with a significantly lower risk for EVD-RI.

**Discussion and conclusion:**

EVD-RI risk is driven primarily by periprocedural rather than patient-related factors. Avoiding EVD exchanges, preventing leaks/obstruction, minimizing drainage duration, and early definitive diversion may reduce infections.

## Highlights

• Purposeful Cox modelling identified independent predictors without confounding.
• Confirmed EVD-RI occurred in 16.3% of patients.
• Periprocedural factors, not comorbidity, primarily drove EVD-RI risk.
• EVD exchange and subarachnoid hemorrhage increased infection risk.
• Postoperative systemic antibiotics and additional neuromonitoring reduced EVD-RI.

## 1 Introduction

The placement of external ventricular drains is among the most frequently performed neurosurgical procedures, particularly in the setting of neurocritical care (Muralidharan, 2015). The procedure was described as early as 1744 by Claude-Nicholas Le Cat (Srinivasan et al., 2014). Since then, various aspects of external ventricular drains (EVDs) including indications, surgical technique, and catheter design have continuously evolved (Srinivasan et al., 2014).

Although routinely performed in neurosurgical centers worldwide, external ventricular drainage is associated with important limitations and complications (Gu et al., 2025). Most importantly, EVDs are not a permanent solution in patients requiring mid-to long-term cerebrospinal fluid (CSF) diversion (Akinduro et al., 2020). Patients who develop persistent hydrocephalus require conversion to a ventriculoperitoneal (VP) or ventriculoatrial (VA) shunt (Akinduro et al., 2020). These conversions to VP and VA shunts are typically performed a few weeks after initial EVD placement, with EVD indwelling time rarely exceeding 30 days (Gu et al., 2025; Nelson et al., 2022). Reported postoperative complications in EVD implantation include catheter malfunction, drainage obstruction, hemorrhage, malposition, and superficial wound infection. The predominant complication, though, is EVD-related infection (EVD-RI), which occurs, the longer the catheter (and thus the fistula to the skin microbiome) is left in place (Muralidharan, 2015; Gu et al., 2025).

Over the past two decades, numerous studies have investigated the incidence of EVD-RIs, with reported EVD-RI rates ranging from under 5% to 30% (approximately 5 to 20 EVD-RIs per 1000 catheter days) (Sweid et al., 2021; Kim et al., 2012, 2022; Walek et al., 2022; Dos Santos et al., 2017; Woo et al., 2017; Mounier et al., 2019; Wang et al., 2023). Beyond infection rates, few studies have sought to identify risk factors predisposing to EVD-RI (Zhou et al., 2023). The reported risk factors differ substantially across studies, largely due to heterogeneity in study design, patient populations, and - most importantly - the definition of EVD-RI applied (Zhou et al., 2023). While multiple studies have attempted to identify risk factors, only a few provide large case series and analysis of multiple parameters with properly adjusted, multivariable methods.

This study aimed to further contribute to this topic by analyzing patient-related risk factors for EVD-RI using a survival modeling approach, adjusting for relevant parameters using the “purposeful variable selection” algorithm described by Hosmer and Lemeshow (Hosmer et al., 2013).

## 2 Methods

### 2.1 Study design and patient selection

This monocentric cohort study – combining data from our prospective patient registry with retrospective chart review – was conducted at the Department of Neurosurgery, University Hospital Zurich. Ethical approval for the institutional research registry (KEK-ZH-PB-2017-00093) had been previously granted, and data for the present analysis were obtained from this registry. The requirement for informed consent was waived. Adult patients (≥18 years) who underwent EVD implantation at our institution between 2014 and 2022 were eligible for inclusion. Patients were excluded if clinical data was incomplete, or postoperative CT imaging was missing.

### 2.2 EVD placement and management

EVDs were inserted by neurosurgeons under sterile conditions, either in the operating theater or within the neurocritical care unit. EVDs were placed using a right frontal linear or curvilinear incision and tunneled for more than 5 cm subcutaneously. Standard practice involved the use of Bactiseal® catheter systems coated with 0.15% Clindamycin and 0.054% Rifampicin (Integra Lifesciences, Princeton, NJ, USA). A single perioperative dose of cefuroxime 1.5g or 3g depending on body weight was administered as antibiotic prophylaxis. Non-impregnated sterile dressings were used to cover the wound postoperatively and changed every two days in accordance with institutional standard. Routine CSF monitoring includes sampling twice per week, with additional collections obtained if EVD-RI is suspected. CSF collection was carried out directly from the EVD under sterile precautions. The first 2 ml were discarded, and the following 3 ml were used for analysis.

### 2.3 Data collection

Patient-related variables (expect Charlston Comorbidity Index (CCI) as well as its single components) and periprocedural variables (except catheter- and infection-related variables) were extracted from the institutional prospective registry. CCI, its single components, catheter- and infection-related variables (catheter indwelling time, catheter leak and catheter exchange) had to be collected retrospectively from electronic medical records as they were not routinely recorded in the prospective registry.

Patient-related factors included demographic information (age, sex), Glasgow Coma Scale (GCS) at admission, CCI at admission as well as its single components (smoking, obesity, dyslipidemia, diabetes mellitus, heart failure, myocardial infarction, peripheral vascular disease, stroke, pulmonary disease, rheumatic disease, liver disease, renal disease, solid tumors, and metastatic tumors), and immunosuppression at admission. Furthermore, periprocedural factors including the underlying pathology, presence of intraventricular hemorrhage, angiography performed, additional hemicraniectomy, need for neuromonitoring, administration of alteplase, additional treatment modality for underlying pathology (surgical procedure, interventional procedure), administration of postoperative antibiotics due to another infection (not EVD-RI), rebleeding within 24 h, occurrence of early (≤7 days postoperatively) or late (7 > days postoperatively) catheter leak, non-EVD-RI-related catheter exchange (due to EVD blockage) and time to catheter removal. In case of CSF infection, the time from admission to infection was documented.

### 2.4 Outcome measures

In this study, the primary outcome was defined as *suspected* EVD-RI leading to systemic antibiotic administration. Suspected EVD-RI were defined as having at least two of the following three conditions based on the recommendations of the Infectious Diseases Society of America (IDSA) and a study by Pietrzko et al. investigating diagnostic workup and antibiotic administrations in cases of EVD-RI:

1. White blood count (WBC) in CSF >500/μl (Tunkel et al., 2017; Pietrzko et al., 2024).
2. Elevated systemic inflammatory parameters: C-reactive protein [CRP] >5 mg/l, Procalcitonin [CRP] > 0.1 μg/l or WBC >9.6G/l after exclusion of any alternative cause (Tunkel et al., 2017; Pietrzko et al., 2024).
3. Clinical signs of EVD-RI: fever (≥38.3 °C when measured in the ear or bladder; 38.0 °C when measured intracerebrally), meningeal irritation, unclear neurological deterioration not attributable explained by an alternative cause (Tunkel et al., 2017; Pietrzko et al., 2024).

Confirmed EVD-RI was defined as a positive CSF culture and/or broad-range eubacterial PCR (ePCR) with concurrent clinical suspicion, as suggested by Pietrzko et al. (2024) Secondary outcomes included the identification of risk factors for suspected EVD-RI and the assessment of clinical outcomes associated with EVD-RI.

### 2.5 Statistical analysis

Continuous variables were summarized as mean ± standard deviation, and categorical variables as counts and percentages. A three-step multivariable purposeful variable selection method applied onto a multivariable Cox proportional hazard model was used for identifying independent predictors and confounders for suspected EVD-RI, as suggested by Hosmer and Lemeshow and explained in detail by Bursac et al. (Hosmer et al., 2013; Bursac et al., 2008) In the first step, variables with *p* < 0.25 in the univariable analysis were included. In the second step, independent predictors were retained at *p* < 0.1, while variables with *p* < 0.25 were considered potential confounders and evaluated for whether they produced a >20% change in the coefficient of any remaining variable, thereby qualifying as confounders. In the third step, all variables identified as neither predictors nor confounders were reintroduced one by one and tested for their influence; those causing a >20% change in the coefficient of any remaining variable were also classified as confounders. A final multivariable Cox model was then built and reported, including all variables (independent predictors and confounders) identified by the purposeful variable selection algorithm. In the final model, two-tailed p < 0.05 was seen as statistically significant. Survival data were right-censored. Kaplan-Meier plots with survival tables are provided along with 95% confidence intervals (CI). All statistical analyses and graph generation were performed using R version 4.2.3 (R Foundation for Statistical Computing, Vienna, Austria).

## 3 Results

### 3.1 Patient characteristics

Detailed patient characteristics are provided in Table 1. A total of 387 patients with 421 EVDs, corresponding to 9538 catheter days, were included in this study. The mean age at admission was 60.2 ± 14.0 years, and 53.2% were female. Postoperative antibiotics for any suspected infection were administered to 69.5% (n = 269) of patients. aSAH was the most common underlying pathology, accounting for 61.5% (n = 238) of cases. 22.2% (n = 86) of patients underwent an additional surgical procedure (aneurysm clipping, tumor extirpation) and 36.2% (n = 140) additional endovascular procedures (aneurysm coiling, fistula embolization), while in 41.6% (n = 161) of patients no additional intervention was performed. An additional hemicraniectomy was performed on 9.8% (n = 38) of patients. Rebleeding of the aneurysm/hemorrhage after EVD implantation occurred in 5.9% (n = 23). A catheter leak was documented in 16.3% (n = 63), the majority being late leaks (n = 57/63; 90.5%). At admission, the mean Glasgow Coma Scale (GCS) score was 9.9 ± 4.7, and the mean Charlson Comorbidity Index (CCI) was 2.6 ± 2.4. The mean duration of catheterization was 18.7 ± 15.6 days.

**Table 1:** Summary of parameters investigated for significant association with EVD-RI risk.

Parameter	Total (n = 387)	No VRI (n = 283)	VRI (n = 104)
Patient female sex, n (%)	206 (53.2)	155 (54.8)	51 (49.0)
Patient age [y], mean (SD)	60.17 (14.0)	61.22 (14.1)	57.29 (13.6)
GCS at admission, mean (SD)	9.86 (4.7)	9.38 (4.8)	11.14 (4.3)
CCI at admission, mean (SD)	2.60 (2.4)	2.81 (2.5)	2.04 (2.0)
Comorbidities			
Hypertension, n (%)	168 (43.4)	132 (46.6)	36 (34.6)
Obesity, n (%)	16 (4.1)	13 (4.6)	3 (2.9)
Dyslipidemia, n (%)	43 (11.1)	31 (11.0)	12 (11.5)
Heart failure, n (%)	18 (4.7)	16 (5.7)	2 (1.9)
Myocardial infarction, n (%)	24 (6.2)	17 (6.0)	7 (6.7)
Peripheral vascular disease, n (%)	16 (4.1)	15 (5.3)	1 (1.0)
Stroke, n (%)	23 (5.9)	21 (7.4)	2 (1.9)
Lung disease, n (%)	38 (9.8)	28 (9.9)	10 (9.6)
Rheumatic disease, n (%)	17 (4.4)	16 (5.7)	1 (1.0)
Kidney disease, n (%)	10 (2.6)	9 (3.2)	1 (1.0)
Solid tumor, n (%)	23 (5.9)	17 (6.0)	6 (5.8)
Metastatic tumor, n (%)	15 (3.9)	12 (4.2)	3 (2.9)
Smoking, n (%)	76 (19.6)	54 (19.1)	22 (21.2)
Immunosuppression at admission, n (%)	17 (4.4)	14 (4.9)	3 (2.9)
Liver disease, n (%)			
Mild	7 (1.8)	6 (2.1)	1 (1.0)
Moderate or severe	9 (2.3)	7 (2.5)	2 (1.9)
Diabetes, n (%)			
Mild	25 (6.5)	20 (7.1)	5 (4.8)
Moderate or severe	4 (1.0)	4 (1.4)	0 (0.0)
Intraventricular hemorrhage, n (%)	302 (78.0)	221 (78.1)	81 (77.9)
Underlying pathology, n (%)			
Intracerebral bleeding	87 (22.5)	74 (26.1)	13 (12.5)
Aneurysmal subarachnoid hemorrhage	238 (61.5)	167 (59.0)	71 (68.3)
Non-aneurysmal subarachnoid hemorrhage	26 (6.7)	17 (6.0)	9 (8.7)
Arteriovenous malformation	22 (5.7)	17 (6.0)	5 (4.8)
Arteriovenous fistula	11 (2.8)	8 (2.8)	3 (2.9)
Other	3 (0.8)	0 (0.0)	2 (1.9)
Treatment type for intracranial hemorrhage, n (%)			
No intervention	161 (41.6)	131 (46.3)	30 (28.8)
Additional surgical procedure	140 (36.2)	92 (32.5)	48 (46.2)
Additional interventional procedure	86 (22.2)	60 (21.2)	26 (25.0)
Hemicraniectomy, n (%)	38 (9.8)	27 (9.5)	11 (10.6)
Neuromonitoring, n (%)	62 (16.0)	47 (16.6)	15 (14.4)
Angiography, n (%)	249 (6.4)	169 (59.7)	80 (76.9)
Administration of alteplase, n (%)	15 (3.9)	12 (4.2)	3 (2.9)
Postoperative antibiotic treatment, n (%)	269 (69.5)	204 (72.1)	65 (62.5)
Rebleeding, n (%)	23 (5.9)	14 (4.9)	9 (8.7)
Catheter indwelling time [d], mean (SD)	18.74 (15.6)	22.17 (16.4)	9.47 (7.0)
Catheter leak (any timepoint), n (%)	63 (16.3)	31 (11.0)	32 (30.8)
Early catheter leak, n (%)	6 (1.6	4 (1.4)	2 (1.9)
Late catheter leak, n (%)	57 (14.7)	27 (9.5)	30 (28.8)
Catheter exchange, n (%)	66 (17.1)	31 (11.0)	35 (33.7)

SD, standard deviation; EVD-RI, external ventricular drain-related infection; GCS, Glasgow coma scale; CCI, Charleston Comorbidity Index; EVD, external ventricular drain.

### 3.2 EVD-RI incidence

The cumulative incidence at defined post-insertion dates is summarized in Table 2. Of the 387 patients included in the analysis, 283 (73.1%) did not develop CSF infection, whereas 104 (26.9%) received antibiotic treatment for suspected EVD-RI. Among these, microbiological confirmation of CSF infection by either positive CSF culture or ePCR was obtained in 63 patients (16.3%). Considering that a total of 421 EVD implantations were performed in the 387 patients, this corresponds to 24.7% of all implanted catheters being associated with suspected EVD-RI and 15.0% with confirmed EVD-RI.

**Table 2:** Cumulative incidence of EVD-RI and number at risk.

Days indwelling	Cumulative EVD-RI incidence, n (%)	Number at risk, n (%)
Day of insertion	0 (0.00)	387 (100.00)
3	7 (1.81)	351 (90.70)
6	43 (11.11)	301 (77.77)
**7**	**56 (14.47)**	**286 (73.90)**
9	73 (18.86)	251 (64.86)
12	82 (21.19)	224 (57.88)
**14**	**86 (22.22)**	**208 (53.75)**
15	88 (22.74)	201 (51.94)
18	94 (24.29)	175 (45.22)
**21**	**97 (25.06)**	**146 (37.73)**
24	99 (25.58)	120 (31.01)
27	100 (25.84)	99 (25.58)
30	101 (26.09)	85 (21.96)

EVD-RI, external ventricular drain-related infection.

### 3.3 Purposeful variable selection model

**Fig. 1:**
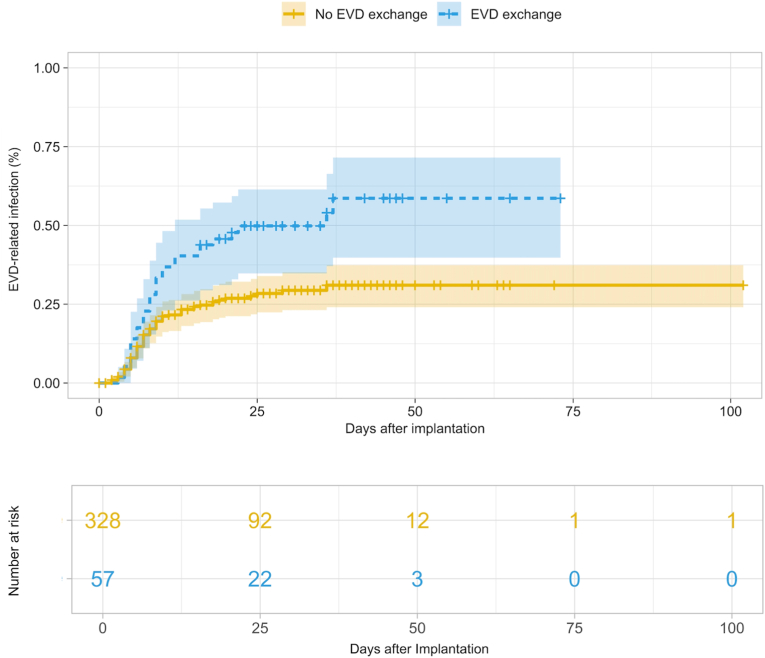
Kaplan-Meier curve showing external ventricular drain-related infection rates in patients with vs. without catheter exchange. Data were right-censored.

**Fig. 2:**
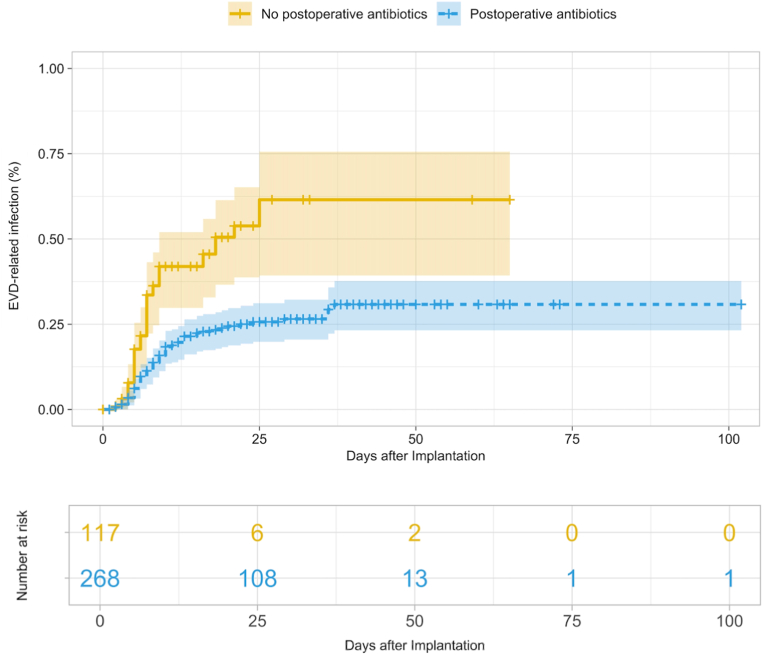
Kaplan-Meier curve showing external ventricular drain-related infection rates in patients with vs. without postoperative antibiotic treatment (postoperative indicating additional antibiotics administered after the prophylactic intraoperative dose, given for infections other than suspected/confirmed EVD-RI, e.g. pneumonia, urinary tract infections). Data were right-censored.

In the purposeful variable selection, EVD exchange (HR: 3.13, 95% confidence interval (CI): 2.02-4.85, *p* < 0.001) (Fig. 1), aneurysmal subarachnoid hemorrhage (aSAH) (HR: 2.56, 95% CI: 1.40-4.68, *p* = 0.002), and non-aneurysmal SAH (HR: 2.11, 95% CI: 0.90-4.96, *p* = 0.09) were associated with a significantly higher risk, whereas postoperative systemic antibiotics given for other suspected infections (HR: 0.33, 95% CI: 0.21-0.51, *p* < 0.001) (Fig. 2) and additional implantation of neuromonitoring (HR: 0.54, 95% CI: 0.30-0.97, *p* = 0.04) were associated with a significantly lower risk for EVD-RI. According to the predefined threshold of >20% change in coefficients, none of the variables qualified as a confounder. None of the comorbidity factors were confounding or independently predictive of EVD-RI. These results are also demonstrated in Table 3.

**Table 3:** Independent Predictors identified in the Purposeful Variable Selection Model. Data were right censored.

Parameter	Hazard ratio	LCL	UCL	*p*-value
Postoperative antibiotics	0.33	0.21	0.51	<0.001
EVD exchange	3.13	2.02	4.85	<0.001
Neuromonitoring	0.54	0.30	0.97	0.04
Indication for EVD placement				
Intracerebral bleeding	Reference			
Aneurysmal subarachnoid hemorrhage	2.56	1.40	4.68	0.002
Non-aneurysmal subarachnoid hemorrhage	2.11	0.90	4.96	0.09
Arteriovenous malformation	0.98	0.34	2.78	0.96
Arteriovenous fistula	1.15	0.32	4.08	0.83
Other	2.03	0.55	7.60	0.29

LCL, lower confidence limit; UCL, upper confidence limit; EVD, external ventricular drain.

## 4 Discussion

In this retrospective study, patient-related risk factors were investigated for the development of EVD-RIs in patients following EVD placement. It is the first study to apply the purposeful variable selection method proposed by Hosmer and Lemeshow within a multivariable Cox proportional hazards model onto a big patient population with the aim of identifying risk factors for EVD-RI (Hosmer et al., 2013). While prior studies (mostly with generalized linear models) have analyzed risk factors for EVD-RI, the increasing incidence over time makes survival modeling a far more powerful and appropriate statistical method. On the other hand, proper selection of adjustment covariates is crucial. For example, EVD exchange is largely dependent on presence of CSF leaks – this just being one example of why poor adjustment can lead to faulty conclusions. The purposeful variable selection algorithm is optimal because it includes both strong independent predictors as well as weakly and indirectly associated confounding variables, which is crucial.

Our analysis indicated that patient comorbidity had only a negligible effect on infection risk in the purposeful variable selection model, whereas patient-related periprocedural treatment decisions having a significantly affected EVD-RI risk. This suggests that there is an intrinsic infection risk over time as long as the fistula to the external skin microbiome is maintained, independent of the patient's comorbidities. In contrast to many other healthcare-associated infections, where systemic conditions such as diabetes are almost invariably strong predictors, EVD-RI risk appears to be driven primarily by the persistent exposure of the ventricular system to the cutaneous microbiome (Tomic et al., 2022). Moreover, there was a considerably higher rate of suspected EVD-RIs compared to confirmed EVD-RIs, surpassing the reported incidences of EVD-RIs in most studies - a finding in line with the discrepancy described by Sohn et al. (2022)

### 4.1 Definition of EVD-RI

The definition of EVD-RI strongly influences both reported incidence and identified risk factors (Woo et al., 2017; Sohn et al., 2022). Several groups have proposed standardized definitions, but no international consensus exists (Woo et al., 2017; Freeman et al., 2019; Gozal et al., 2014; Lewis et al., 2016). Many studies rely solely on microbiological confirmation, thereby avoiding false-positive clinical suspicions but overlooking the fact that antibiotics are usually started empirically (Woo et al., 2017). Conversely, low bacterial loads may yield false-negative cultures, leading to missed cases (Sohn et al., 2022). As Sohn et al. emphasized, improved microbiological diagnostics and novel biomarkers are required to ensure accurate discrimination (Sohn et al., 2022).

In this study, 26.9% of patients had clinically suspected EVD-RI, whereas only 16.4% had microbiologically confirmed EVD-Ri, underlining the differences in incidence depending on EVD-RI definition. Although the confirmed rate remains in the upper third compared to previous reports, this can be explained by our relatively loose definition: every positive CSF culture or ePCR result was considered diagnostic (apart from clear presence of contamination with skin commensals without need for antibiotic therapy), whereas other studies required positive cultures plus clinical signs and CSF changes (Gozal et al., 2014; Lewis et al., 2016). Our approach, defining suspected EVD-RI as the presence of at least two IDSA-based clinical, laboratory, or inflammatory criteria and considering confirmed EVD-RI only when supported by positive CSF culture or ePCR, combines clinical relevance with diagnostic accuracy, thereby reducing both false-positive and false-negative cases. Moreover, the integration of broad-range ePCR provides a particularly sensitive diagnostic tool, capable of detecting infections with low bacterial loads that might otherwise be missed by conventional culture (Pietrzko et al., 2024).

### 4.2 Independent predictors and clinical implications

#### 4.2.1 Catheter exchange

The need for (non-EVD-RI-related) catheter exchange was an identified independent predictor for heightened EVD-RI risk in this study, a finding in line with previous studies (Sweid et al., 2021; Zhou et al., 2023; Katzir et al., 2019; Lo et al., 2007). Possible explanations for that are that manipulation of the ventricular system may introduce pathogens, and the need for exchange typically reflects prolonged drainage duration, itself a known risk factor (Sweid et al., 2021; Kim et al., 2012, 2022; Bota et al., 2005; Arabi et al., 2005). This finding argues against routine or prophylactic replacement and favors an exchange-sparing strategy with minimal manipulation of the closed system.

#### 4.2.2 Underlying pathology

Aneurysmal and non-aneurysmal SAH were identified as independent predictors of increased EVD-RI risk. This is consistent with the findings of Zhou et al., who reported any form of SAH as a significant risk factor (Zhou et al., 2023). Furthermore, Ebel et al. demonstrated that both the extent and anatomical location of SAH play a decisive role: in particular, a larger subarachnoid blood clot and localization in the posterior fossa were significantly associated with higher EVD-RI risk (Ebel et al., 2025).

#### 4.2.3 Postoperative systemic antibiotics

Having had postoperative administration of antibiotics due to other suspected infections such as pneumonia or urinary tract infections was another factor identified as significantly reducing the risk for EVD-RI. These findings go hand in hand with the findings of Murphy et al. outlining that short-term perioperative systemic antibiotic treatment in combination with antibiotic-coated catheters does decrease EVD-RI, simultaneously showing that prolonged systemic antibiotic treatment is not of benefit (Murphy et al., 2015). Clinically, this would in theory support ensuring preventive antibiotic coverage during high-risk windows - early after implantation and around manipulations or exchanges - while avoiding blanket prophylaxis and adhering to antimicrobial-stewardship principles.

#### 4.2.4 Neuromonitoring

To the best of our knowledge, no previous study has examined whether the insertion of neuromonitoring is associated with EVD-RI risk. In our cohort, additional implantation of neuromonitoring was found to reduce the risk of EVD-RI. Since neuromonitoring is typically applied in patients with particularly severe pathology, one would expect these patients to have a higher risk of EVD-RI due to prolonged drainage times (Stocchetti et al., 2013). However, a possible explanation for this finding is that these patients receive more postoperative antibiotics for concomitant complications, e.g. aspiration pneumonia. As postoperative antibiotic treatment has been found to be significantly associated with lower EVD-RI risk in this study, this could account for the paradoxical protective association observed. Clinically, this explanation would further support preventive antibiotic during high-risk windows. However, this finding should be interpreted with caution, and further studies are required to evaluate the role of neuromonitoring in the context of EVD; accordingly, it should not be taken as a clinical recommendation for infection prevention.

### 4.3 Limitations

Several limitations must be considered in the context of this study. First, although the analysis was predominantly based on a prospective registry, additional data were collected retrospectively. Limitations therefore mainly apply to the retrospective component, where selection bias and missing data may have influenced the results, particularly as patients with incomplete data had to be excluded in a complete case analysis. Second, the generalizability of our findings is limited by institutional practices. For example, our standardized protocol mandates routine CSF sampling twice weekly. While this consistency precluded us from analyzing the frequency of CSF sampling as a risk factor, it also means that results may differ in centers with different or less standardized approaches, as illustrated by published bundle protocols where CSF sampling is often performed less regularly (Rojas-Lora et al., 2023). Another example for different institutional practices lies in postoperative wound management, as non-impregnated sterile dressings were used to cover the wound postoperatively. A recent study by Stohler et al. however suggests that chlorhexidine-containing cutaneous dressings can reduce EVD-RI (Stohler et al., 2025). Third, although diagnostic criteria for EVD-RI were applied consistently and in line with IDSA recommendations, they inevitably remain influenced by institutional judgment in the absence of universally accepted standards. Fourth, surgeon- and surgery-related variables, such as surgeon experience, duration of surgery, insertion location, tunnelling length and incision type have not been analyzed in this study. Finally, although the sample size was sizeable and adequate for the present analysis, a larger sample might have provided greater statistical power to detect smaller effect sizes, which could remain undetected in our dataset.

## 5 Conclusions

This study demonstrates that EVD-RI risk is driven primarily by periprocedural factors rather than patient comorbidity. Independent predictors heightening EVD-RI risk included catheter exchange and subarachnoid hemorrhage, while postoperative systemic antibiotics and neuromonitoring were associated with reduced risk. Preventive strategies should therefore prioritize exchange-sparing approaches, early definitive CSF diversion when feasible, and refined diagnostic and antimicrobial protocols to reduce infection burden. Furthermore, the discrepancy between suspected and confirmed infections underscores the urgent need for harmonized diagnostic criteria.

## Availability of data and material

The data in support of our findings can be obtained upon reasonable request from the corresponding author.

## Funding

This research did not receive any specific grants from funding agencies in the public, commercial, or not-for-profit sectors.

## Conflict of interest

The authors declare that the article and its contents were composed in the absence of any commercial or financial relationships that could be construed as a potential conflict of interest.

## References

[bib1] Akinduro O.O., Vivas-Buitrago T.G., Haranhalli N., Ganaha S., Mbabuike N., Turnbull M.T., Tawk R.G., Freeman W.D. (2020). Predictors of Ventriculoperitoneal shunting following Subarachnoid Hemorrhage treated with external ventricular drainage. Neurocritical Care.

[bib2] Arabi Y., Memish Z.A., Balkhy H.H., Francis C., Ferayan A., Al Shimemeri A., Almuneef M.A. (2005). Ventriculostomy-associated infections: incidence and risk factors. Am. J. Infect. Control.

[bib3] Bota D.P., Lefranc F., Vilallobos H.R., Brimioulle S., Vincent J.-L. (2005). Ventriculostomy-related infections in critically ill patients: a 6-year experience. J. Neurosurg..

[bib4] Bursac Z., Gauss C.H., Williams D.K., Hosmer D.W. (2008). Purposeful selection of variables in logistic regression. Source Code Biol. Med..

[bib5] Dos Santos S.C., Fortes Lima T.T., Lunardi L.W., Stefani M.A. (2017). External ventricular drain-related infection in spontaneous intracerebral hemorrhage. World Neurosurg..

[bib6] Ebel F., Westarp E., Poretti M., von Rotz M., Stohler S., Chen R., Guzman R., Weisser M., Tschudin-Sutter S., Mariani L., Roethlisberger M. (2025). Impact of hemorrhage extent on external ventricular drain-associated infections in aneurysmal subarachnoid hemorrhage. Neurocritical Care.

[bib7] Freeman W.D., Ziai W.C., Hanley D. (2019). Ventriculostomy-associated infection (VAI): in search of a definition. Neurocritical Care.

[bib8] Gozal Y.M., Farley C.W., Hanseman D.J., Harwell D., Magner M., Andaluz N., Shutter L. (2014). Ventriculostomy-associated infection: a new, standardized reporting definition and institutional experience. Neurocritical Care.

[bib9] Gu C., Lind A.N.R., Haldrup M., Eschen J.T., Eskildsen M.H., Kjær A., Rasmussen M., Dyrskog S., Meier K., Simonsen C.Z., Debrabant B., Korshøj A.R. (2025). Outcomes and complications of external ventricular drainage in primary and secondary intraventricular hemorrhage: a descriptive observational study. J. Neurosurg..

[bib10] Hosmer D.W., Lemeshow S., Sturdivant R.X. (2013).

[bib11] Katzir M., Lefkowitz J.J., Ben-Reuven D., Fuchs S.J., Hussein K., Sviri G.E. (2019). Decreasing external ventricular drain-related infection rates with duration-independent, clinically indicated criteria for drain revision: a retrospective study. World Neurosurg..

[bib12] Kim J.-H., Desai N.S., Ricci J., Stieg P.E., Rosengart A.J., Härtl R., Fraser J.F. (2012). Factors contributing to ventriculostomy infection. World Neurosurg..

[bib13] Kim J., Kim J.H., Lee W., Han H.J., Park K.Y., Chung J., Kim Y.B., Joo J.Y., Park S.K. (2022). Predictors of ventriculostomy-associated infections: a retrospective study of 243 patients. World Neurosurg..

[bib14] Lewis A., Wahlster S., Karinja S., Czeisler B.M., Kimberly W.T., Lord A.S. (2016). Ventriculostomy-related infections: the performance of different definitions for diagnosing infection. Br. J. Neurosurg..

[bib15] Lo C.H., Spelman D., Bailey M., Cooper D.J., Rosenfeld J.V., Brecknell J.E. (2007). External ventricular drain infections are independent of drain duration: an argument against elective revision. J. Neurosurg..

[bib16] Mounier R., Birnbaum R., Cook F., Jost P.-H., Martin M., Aït-Mamar B., Nebbad B., Couffin S., Tomberli F., Djedid R., Dhonneur G., Lobo D. (2019). Natural history of ventriculostomy-related infection under appropriate treatment and risk factors of poor outcome: a retrospective study. J. Neurosurg..

[bib17] Muralidharan R. (2015). External ventricular drains: management and complications. Surg. Neurol. Int..

[bib18] Murphy R.K.J., Liu B., Srinath A., Reynolds M.R., Liu J., Craighead M.C., Camins B.C., Dhar R., Kummer T.T., Zipfel G.J. (2015). No additional protection against ventriculitis with prolonged systemic antibiotic prophylaxis for patients treated with antibiotic-coated external ventricular drains. JNS.

[bib19] Nelson S.E., Suarez J.I., Sigmon A., Hua J., Weiner C., Sair H.I., Stevens R.D. (2022). External ventricular drain use is associated with functional outcome in aneurysmal subarachnoid hemorrhage. Neurol. Res. Pract..

[bib20] Pietrzko E., Bögli S., Frick K., Ebner-Dietler S., Capone C., Imkamp F., Koliwer-Brandl H., Müller N., Keller E., Brandi G. (2024). Broad range eubacterial polymerase chain reaction of cerebrospinal fluid reduces the time to exclusion of and costs associated with ventriculostomy-related infection in hemorrhagic stroke. Neurocritical Care.

[bib21] Rojas-Lora M., Corral L., Zabaleta-Carvajal I., López-Ojeda P., Fuentes-Mila V., Romera-Peregrina I., Lerma-Briansò C., Plata-Menchaca E., Pavón A., Sabater J., Cabellos C. (2023). External ventriculostomy-associated infection reduction after updating a care bundle. Ann. Clin. Microbiol. Antimicrob..

[bib22] Sohn S.Y., Russell C.D., Jamjoom A.A.B., Poon M.T., Lawson McLean A., Ahmed A.I. (2022). British neurosurgical Trainee research collaborative, comparison of suspected and confirmed internal external ventricular drain-related infections: a prospective multicenter United Kingdom observational study. Open Forum Infect. Dis..

[bib23] Srinivasan V.M., O'Neill B.R., Jho D., Whiting D.M., Oh M.Y. (2014). The history of external ventricular drainage. J. Neurosurg..

[bib24] Stocchetti N., Le Roux P., Vespa P., Oddo M., Citerio G., Andrews P.J., Stevens R.D., Sharshar T., Taccone F.S., Vincent J.-L. (2013). Clinical review: neuromonitoring - an update. Crit. Care.

[bib25] Stohler S., Giudice E., Von Rotz M., Ebel F., Westarp E., Poretti M., Chen R., Cueni N., Widmer A.F., Mariani L., Tschudin-Sutter S., Weisser-Rohacek M., Moffa G., Roethlisberger M. (2025). Long-term efficacy of chlorhexidine-containing cutaneous dressings on ventriculostomy-related infection: a 10-year before-and-after study. Neurosurg. Focus.

[bib26] Sweid A., Weinberg J.H., Abbas R., El Naamani K., Tjoumakaris S., Wamsley C., Mann E.J., Neely C., Head J., Nauheim D., Hauge J., Gooch M.R., Herial N., Zarzour H., Alexander T.D., Missios S., Hasan D., Chalouhi N., Harrop J., Rosenwasser R.H., Jabbour P. (2021). Predictors of ventriculostomy infection in a large single-center cohort. J. Neurosurg..

[bib27] Tomic D., Shaw J.E., Magliano D.J. (2022). The burden and risks of emerging complications of diabetes mellitus. Nat. Rev. Endocrinol..

[bib28] Tunkel A.R., Hasbun R., Bhimraj A., Byers K., Kaplan S.L., Scheld W.M., van de Beek D., Bleck T.P., Garton H.J.L., Zunt J.R. (2017). Infectious diseases society of America's Clinical practice guidelines for healthcare-associated ventriculitis and Meningitis. Clin. Infect. Dis..

[bib29] Walek K.W., Leary O.P., Sastry R., Asaad W.F., Walsh J.M., Horoho J., Mermel L.A. (2022). Risk factors and outcomes associated with external ventricular drain infections. Infect. Control Hosp. Epidemiol..

[bib30] Wang P., Luo S., Cheng S., Gong M., Zhang J., Liang R., Ma W., Li Y., Liu Y. (2023). Construction and validation of infection risk model for patients with external ventricular drainage: a multicenter retrospective study. Acta Neurochir (Wien).

[bib31] Woo P.Y.M., Wong H.-T., Pu J.K.S., Wong W.-K., Wong L.Y.W., Lee M.W.Y., Yam K.-Y., Lui W.-M., Poon W.-S. (2017). Moving the goalposts: a comparison of different definitions for primary external ventricular drain infection and its risk factors: a multi-center study of 2575 patients. J. Clin. Neurosci..

[bib32] Zhou J., Zhong Y., Li X., Li H., Wang J., Yang S., Chen G. (2023). Risk factors for external ventricular drainage-related infection: a systematic review and meta-analysis. Neurol., Clin. Pract..

